# SET/TAF1 forms a distance-dependent feedback loop with Aurora B and Bub1 as a tension sensor at centromeres

**DOI:** 10.1038/s41598-020-71955-2

**Published:** 2020-09-24

**Authors:** Yuichiro Asai, Rieko Matsumura, Yurina Hasumi, Hiroaki Susumu, Kyosuke Nagata, Yoshinori Watanabe, Yasuhiko Terada

**Affiliations:** 1 Department of Chemistry and Biochemistry, School of Advanced Science and Engineering, Waseda University, 3-4-1 Ohkubo, Shinjuku-ku, Tokyo, 169-8555 Japan; 2 Graduate Program in Biophysics and Biochemistry, Graduate School of Science, The University of Tokyo, Yayoi, Tokyo, 113-0032 Japan; 3 Department of Infection Biology, Faculty of Medicine, University of Tsukuba, 1-1-1 Tennodai, Tsukuba, 305-8575 Japan; 4 Genome Damage and Stability Centre, School of Life Sciences, University of Sussex, Falmer, BN1 9RQ Sussex UK

**Keywords:** Chromosome segregation, Mitotic spindle

## Abstract

During mitosis, spatiotemporal regulation of phosphorylation at the kinetochore is essential for accurate chromosome alignment and proper chromosome segregation. Aurora B kinase phosphorylates kinetochore substrates to correct improper kinetochore-microtubule (KT-MT) attachments, whereas tension across the centromeres inactivates Aurora B kinase, and PP2A phosphatase dephosphorylates the kinetochore proteins to stabilize the attachments. However, the molecular entity of the tension sensing mechanism remains elusive. In a previous report, we showed that centromeric SET/TAF1 on Sgo2 up-regulates Aurora B kinase activity via PP2A inhibition in prometaphase. Here we show that Aurora B and Bub1 at the centromere/kinetochore regulate both kinase activities one another in an inter-kinetochore distance-dependent manner, indicating a positive feedback loop. We further show that the centromeric pool of SET on Sgo2 depends on Bub1 kinase activity, and the centromeric localization of SET decreases in a distance-dependent manner, thereby inactivating Aurora B in metaphase. Consistently, ectopic targeting of SET to the kinetochores during metaphase hyperactivates Aurora B via PP2A inhibition, and thereby rescues the feedback loop. Thus, we propose that SET, Aurora B and Bub1 form a distance-dependent positive feedback loop, which spatiotemporally may act as a tension sensor at centromeres.

## Introduction

Aurora B is a kinase catalytic subunit of the chromosome passenger complex (CPC), that includes three regulatory proteins: inner centromere protein (INCENP), survivin and borealin^1^. During mitosis, Aurora B and PP2A regulate the phosphorylation state of kinetochore proteins spatiotemporally for accurate chromosome alignment and proper chromosome separation. In prometaphase, Aurora B kinase is enriched at the inner centromeres with high activity, and phosphorylates kinetochore substrates to destabilize improper kinetochore-microtubule (KT-MT) attachments^2–4^, whereas the opposing phosphatases PP1 and PP2A accumulate at the kinetochores^5–8^. However, in metaphase, the pulling force by bi-oriented KT-MT attachment increases the centromere–kinetochore distance. The spatial separation model proposes that the increase in the distance pulls Aurora B substrates at the kinetochore out of the sphere of Aurora B kinase activity^9–12^, leading to the reduction of phosphorylation level of kinetochore substrates^4,13,14^. In addition, PP2A is distributed from the centromeres to the kinetochores in metaphase, thereby stabilizing KT-MT attachment by counteracting phosphorylation at the kinetochore^5,15,16^. These mechanisms regulate accurate chromosome alignment.

Furthermore, both the amount of Aurora B and its kinase activity are reported to decrease quickly between stretched and unstretched kinetochores^17,18^. However, the molecular mechanism by which Aurora B kinase activity is regulated in a kinetochore distance-dependent manner remains elusive. It is also unclear whether PP2A activity around centromeres and kinetochores increase from prometaphase to metaphase.

Recently, it is proposed that Aurora B kinase and Bub1 kinase, one of the spindle assembly checkpoint (SAC) proteins, spatiotemporally regulate the localization and activity of one another^17,19–21^. Aurora B regulates SAC activator kinase Mps1 recruitment to kinetochores^22–24^, and Mps1 activates itself through transphosphorylation after clustering^25,26^. Activated Mps1 then phosphorylates the kinetochore protein KNL1 to recruit the Bub1 complex^27–29^. These data suggest that Aurora B regulates the recruitment of Bub1 to kinetochores indirectly. In contrast, Bub1 is also required for the centromeric localization and activation of Aurora B kinase^17,21^. Bub1 phosphorylates histone 2A threonin120 (HH2A pT120) to recruit Shugoshin (Sgo), the scaffold protein of CPC, to the centromere^17,30–32^. Moreover, overexpression of Bub1 hyperactivates Aurora B^33^. These data indicate that Aurora B and Bub1 recruit and activate one another at the centromere and kinetochore, and that these kinases may form a positive feedback loop. In metaphase, however, the latest spatial separation model proposes that the tension-dependent increase in the centromere–kinetochore distance pulls Bub1 at the kinetochore away from the centromere, leading to the removal of Aurora B from centromeres and chromosome bi-orientation^17,34–36^. In contrast, the inactivation of Aurora B in metaphase induces kinetochore localization of PP1, and then PP1 competes with Mps1 to disturb recruitment of Bub1 at the kinetochore, while Aurora B kinase activity suppresses recruitment of PP1 to the kinetochore in prometaphase^6,37^. These data suggest that the positive feedback loop between Aurora B kinase and Bub1 kinase would be attenuated in metaphase by these mechanisms; however, the details remain unclear.

SET/TAF1 forms an inhibitory protein complex with PP2A^38,39^. In a previous report, we showed that SET, a PP2A inhibitor protein, localizes at the centromeres through interaction with a scaffold protein, Sgo2, and maintains Aurora B activity by counteracting PP2A to correct improper KT-MT attachment^40^. Moreover, the centromeric localization of SET with Sgo2 decreases in a kinetochore distance-dependent manner, indicating that the decrease in the centromeric localization of SET ensures the timely formation of stable KT-MT attachment in metaphase. However, the mechanism by which SET is released from the centromere/kinetochore with Sgo2 is not fully known.

In this report we show that Aurora B, Bub1 and SET form a positive feedback loop between inner centromeres and kinetochores. We artificially changed the inter-kinetochore distance of HeLa cells to demonstrate that the amounts of these proteins at the centromere/kinetochore change coordinately in inverse correlation with inter-kinetochore distance from prometaphase to metaphase. We further show that pools of Aurora B and Bub1 remotely regulate each other at the centromere/kinetochore by generating a platform, Bub1-mediated HH2A pT120, for Sgo2 and CPC, which allows the activation of Aurora B kinase by SET on Sgo2. We conclude that the decrease of centromeric localization of SET ensures chromosome bi-orientation by attenuating the positive feedback loop, thereby leading to the activation of PP2A and the inactivation of Aurora B in metaphase.

## Results

### Aurora B and Bub1 coordinately change in inverse correlation with their distance

Aurora B recruits the SAC kinase Mps1 to kinetochores, and clustered Mps1 activates itself through transphosphorylation^25,26^. The activated Mps1 then phosphorylates KNL1 to recruit the Bub1 complex^22,27–29^. Indeed, Bub1 and Bub1-mediated HH2A pT120 signals decrease dramatically in cells treated with the Aurora B kinase inhibitors ZM447439 or AZD1152 (Fig. S1A–D), indicating that Aurora B kinase is required for the localization of Bub1 kinase at the kinetochore. Similarly, Bub1 and its kinase activity are also required for Aurora B and its kinase activity^17,21^, but the localizations of both kinases decrease when tension across the kinetochore pairs is established upon bipolar attachment at metaphase^17,18^.

**Figure 1:**
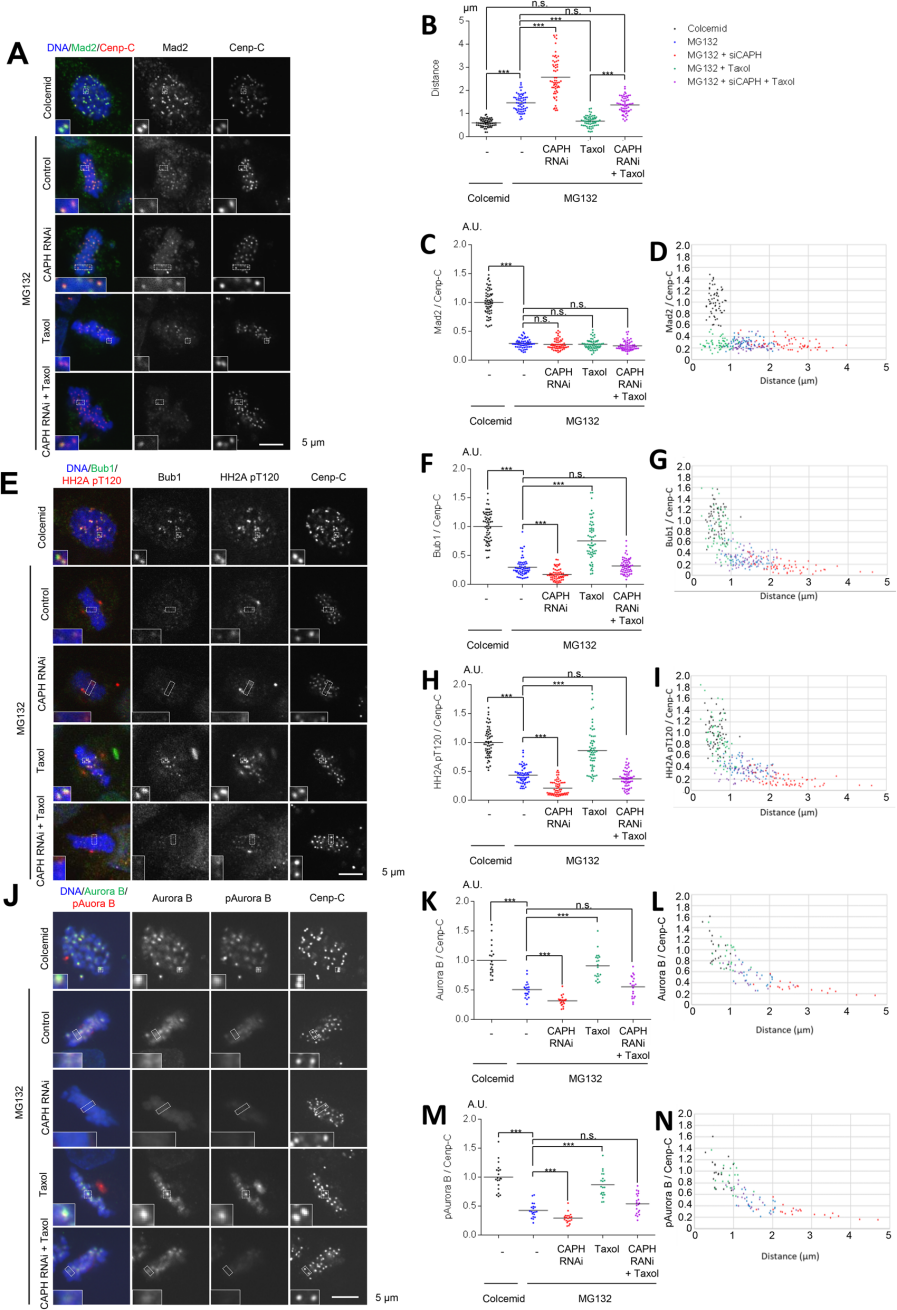
Pools of Aurora B and Bub1 are remotely regulated from the centromere/kinetochore in a kinetochore distance-dependent manner. (**A**–**D**) The kinetochore localization of Mad2 decreases in a kinetochore-microtubule attachment-dependent manner. (**A**) Kinetochore distances were expanded by CAPH RNAi treatment, and shortened by taxol treatment. Time schedules are shown in Fig. S2C, and a schematic diagram is shown in Fig. S2D. HeLa cells treated with siCAPH or siLuciferase (control) were released from RO3306 block and then treated with colcemid (prometaphase) or MG132 (metaphase). After treatment with or without taxol for 30 min, cells were fixed and stained with anti-Mad2 and Cenp-C antibodies. The following 5 conditions with altered kinetochore-distances were prepared as shown in Fig. S2C and D: colcemid treatment (prometaphase), MG132 treatment (metaphase), MG132 + CAPH RNAi treatment (metaphase with expanded kinetochore-distances), MG132 + taxol (metaphase with shortened kinetochore-distances) and MG132 + CAPH RNAi + taxol (kinetochore-distances once expanded by CAPH RNAi treatment were then shortened by taxol treatment). Scale bar, 5 μm. (**B**) The inter-kinetochore distance between paired Cenp-C signals of (**A**) were measured. Bars represent means. Dot plot (N = 60 kinetochore pairs, 3 independent experiment, 5 cells per experiment, 4 kinetochore pairs per cell). (**C**) Relative intensities (Mad2/Cenp-C) of (**A**) are shown. Bars represent means. Dot plot (N = 60 kinetochore pairs, 3 independent experiment, 5 cells per experiment, 4 kinetochore pairs per cell, Mann–Whitney *U*-test, n.s. not significant, ***p < 0.001). (**D**) Relative intensities (Mad2/Cenp-C) of (**A**) are shown versus the distance between kinetochores. Scatter plot. (**E**–**I**) The kinetochore localization of Bub1 decreases in a kinetochore distance-dependent manner. (**E**) HeLa cells synchronized and treated as in (**A**) were stained with Bub1, HH2A pT120 and Cenp-C antibodies. Scale bar, 5 μm. (**F**, **H**) Relative intensities (Bub1/Cenp-C or HH2A pT120/Cenp-C) of (**E**) are shown. Bars represent means. Dot plots (N = 60 kinetochore pairs, 3 independent experiment, 5 cells per experiment, 4 kinetochore pairs per cell, Mann–Whitney *U*-test, n.s. not significant, ***p < 0.001). (**G**, **I**) Relative intensities (Bub1/Cenp-C or HH2A pT120/Cenp-C) of (**E**) are shown versus the distance between kinetochore pairs. Scatter plots. (**J**–**N**) The centromeric localization of Aurora B decreases in a kinetochore distance-dependent manner. (**J**) HeLa cells synchronized and treated as in (**A**) were stained with Aurora B, pAurora B and Cenp-C antibodies. Scale bar, 5 μm. (**K**, **M**) Relative intensities (Aurora B/Cenp-C or pAurora B/Cenp-C) of (**J**) are shown. Bars represent means. Dot plots (N = 20 kinetochore pairs, 5 cells per experiment, 4 kinetochore pairs per cell, Mann–Whitney *U*-test, n.s. not significant, ***p < 0.001). (**L**, **N**) Relative intensities (Aurora B/Cenp-C or pAurora B/Cenp-C) of (**K**) are shown versus the distance between kinetochore pairs. Scatter plots.

To study whether the decrease in the localizations of both kinases in metaphase depends on the inter-kinetochore distance or KT-MT attachment, we artificially regulated the inter-kinetochore distance of metaphase cells. Human cells contain two types of condensin complexes (condensins I and II)^41^, with condensin I controlling the mitosis-specific condensation of chromosomes^42^. Accordingly, it is reported that the depletion of CAPH (chromosome-associated protein H), a component of the condensin I complex, induces the inhibition of mitosis-specific condensation and the increase in the inter-kinetochore distance^43,44^. We treated HeLa cells with siCAPH (siRNA against CAPH) and measured the distance between CENP-C signals by microscopy. CAPH depletion in metaphase-arrested cells resulted in a 2.0-fold increase in kinetochore distance from 1.5 um in control metaphase cells to 3.0 um (Figs. 1A,B, S2A–D). In contrast, taxol treatment, which suppresses microtubule dynamics, decreased the distance between sister kinetochores from 3.0 um to 1.5 um, which is almost the same as in metaphase-arrested control cells (Figs. 1A, B, S2C,D).

We next confirmed the kinetochore localization of Mad2 and Bub1 using the same system. Mad2 strongly localized at the kinetochores only when cells were synchronized in prometaphase using colcemid, a microtubule polymerization inhibitor. To delineate the relationship between inter-kinetochore distance and KT-MT attachment, we treated CAPH-depleted cells with taxol. The amount of Mad2 remained low in metaphase regardless of the kinetochore distance, when siCAPH-cells were treated with or without taxol, clearly indicating that the localization of Mad2 at the kinetochore decreases in a KT-MT attachment-dependent manner (Fig. 1A–D). In contrast, CAPH depletion decreased both Bub1 and HH2A pT120 signals at kinetochores as compared with cells treated with MG132, whereas taxol treatment increased their localization and the histone modification (Fig. 1E–I). These results are consistent with previous reports indicating that Mad2 localization at kinetochores depends on KT-MT attachment^45^, whereas Bub1 localization depends on the inter-kinetochore distance^46^. Accordingly, the amount of Aurora B and the autophosphorylation level of Aurora B at Thr232 (pAurora B) were decreased in the CAPH-depleted cells, but taxol treatment caused them to increase (Fig. 1J–N). These results suggest that Aurora B at the centromere and Bub1 at the kinetochore may recruit and activate each other in a kinetochore distance-dependent manner.

### Both Bub1 and Aurora B kinases are required for recruitment of SET and Sgo2 at centromeres

**Figure 2:**
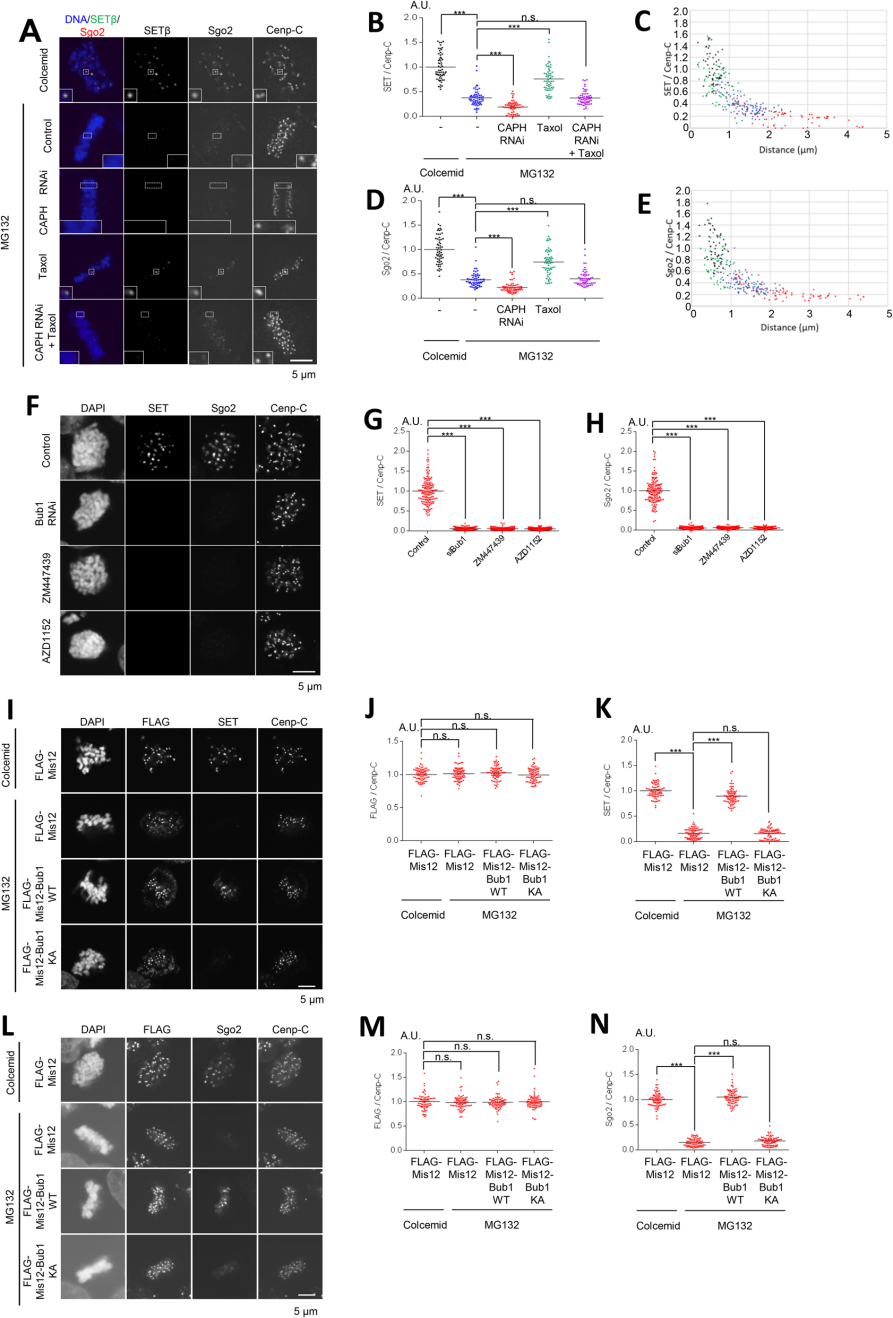
Bub1 and Aurora B kinase are required for the recruitment of SET and Sgo2 to centromeres. (**A**–**E**) The centromeric localizations of SET and Sgo2 decrease in a kinetochore distance-dependent manner as Bub1. (**A**) HeLa cells synchronized and treated as in Fig. 1A were stained with anti-SET, Sgo2 and Cenp-C antibodies. Anti-Sgo2 antibody was used at 1:2,000. Scale bar, 5 μm. (**B**, **D**) Relative intensities (SET/Cenp-C or Sgo2/Cenp-C) of (**A**) are shown. Bars represent means. Dot plots (N = 60 kinetochore pairs, 3 independent experiment, 5 cells per experiment, 4 kinetochore pairs per cell, Mann–Whitney *U*-test, n.s. not significant, ***p < 0.001). (**C**, **E**) Relative intensities (SET/Cenp-C or Sgo2/Cenp-C) of (**A**) are shown versus the distance between kinetochores. Scatter plots. (**F**–**H**) SET and Sgo2 localizations at centromeres were decreased by the depletion of Bub1 kinase or the inhibition of Aurora B kinase activity. (**F**) HeLa cells arrested in prometaphase by colcemid treatment were treated with Bub1 or control RNAi, plus ZM447439 (inhibitor against Aurora kinases), AZD1152 (selective inhibitor against Aurora B kinase) or DMSO (control), and fixed and stained with anti-SET, Sgo2 and Cenp-C antibodies. Anti-Sgo2 antibody was used at 1:5,000. Scale bar, 5 μm. (**G**, **H**) Relative intensities (SET / Cenp-C or Sgo2/Cenp-C) of (**F**) are shown. Bars represent means. Dot plot (N = 75 kinetochore pairs, 3 independent experiment, 5 cells per experiment, 5 kinetochore pairs per cell, Mann–Whitney *U*-test, ***p < 0.001). (**I**–**N**) Expression of Mis12-Bub1 at metaphase restores SET and Sgo2 at inner centromeres. (**I**) HeLa cells expressing 3FLAG-HA-Mis12, 3FLAG-HA-Mis12-Bub1 WT or 3FLAG-HA-Mis12 KA (K821A, kinase dead mutant^47^) were arrested at prometaphase by colcemid treatment or at metaphase by MG132 treatment, and fixed and stained with anti-FLAG, SET and Cenp-C antibodies. Scale bar, 5 μm. (**J**, **K**) Relative intensities (FLAG/Cenp-C or SET/Cenp-C) of (**I**) are shown. Bars represent means. Dot plot (N = 75 kinetochore pairs, 3 independent experiment, 5 cells per experiment, 5 kinetochore pairs per cell, Mann–Whitney *U*-test, ***p < 0.001, n.s. not significant). (**L**) HeLa cells prepared as for (**I**) were fixed and stained with anti-FLAG, Sgo2 and Cenp-C antibodies. Anti-Sgo2 antibody was used at 1:5,000. Scale bar, 5 μm. (**M**, **N**) Relative intensities (FLAG/Cenp-C or Sgo2/Cenp-C) of (**L**) are shown. Bars represent means. Dot plot (N = 75 kinetochore pairs, 3 independent experiment, 5 cells per experiment, 5 kinetochore pairs per cell, Mann–Whitney *U*-test, ***p < 0.001, n.s. not significant).

Our previous study showed the dynamic changes in the localization of both SET and Sgo2 at centromeres during the transition from prometaphase to metaphase: SET co-localizes at inner centromeres with its scaffold protein, Sgo2, but its centromeric localization of aligned chromosomes decreases in metaphase upon bipolar attachment at metaphase^40^. These results suggest that the decrease in the centromeric localization of SET might be required for the establishment of chromosome bi-orientation and proper chromosome segregation. Next, to determine whether localizations of SET and Sgo2 are also similarly modified when the inter-KT distance is altered, we confirmed their localization and dissociation pattern using our assay system. Like Bub1, but not Mad2, both SET and Sgo2 signals were decreased in CAPH-depleted cells treated with MG132 as compared with control metaphase cells, whereas taxol treatment increased these signals (Fig. 2A–E). These results are consistent with the hypothesis that localizations of SET and Sgo2 at centromeres are regulated in a kinetochore distance-dependent manner.

Given that Bub1 is required for the recruitment of Sgo2 at centromeres by phosphorylating histone H2A T120^17,32^, we hypothesized that SET localization is also regulated by Bub1-mediated HH2A pT120. Like Sgo2, SET signals were dramatically decreased at centromeres in prometaphase cells treated with siBub1 (Fig. 2F–H), indicating the possibility that SET localizes at centromeres through direct interaction with Sgo2 on centromeric HH2A pT120 as a scaffold. Since Aurora B and Bub1 are required for the centromere/kinetochore localization of one another (Fig. S1A–D)^17,19–21^, we next examined the possibility that Aurora B kinase activity is also required for SET and Sgo2 localization at centromeres. The signals of both were dramatically decreased at centromeres by treatment with the Aurora B kinase inhibitors ZM447439 or AZD1152 (Fig. 2F–H). These data indicate that SET is recruited to the centromere through Aurora B kinase activity in addition to Bub1 kinase activity, as Sgo2, in consistent with previous reports^19,20,30,40^. Therefore, we hypothesized that localizations of SET and Sgo2 are regulated by the kinetochore distance-dependent feedback loop system that Aurora B and Bub1 form between centromeres and kinetochores. To test this hypothesis, we tethered Bub1 to the metaphase kinetochore by fusing it to the kinetochore protein Mis12 (Mis12-Bub1). Indeed, expression of Mis12-Bub1 WT, but not KA, kinase dead^47^, dramatically restored both SET and Sgo2 signals at centromeres similar to those in prometaphase cells treated with colcemid (Figs. 2I–N and S3A–D). These results suggest that the centromeric localizations of SET and Sgo2 decrease in a kinetochore distance-dependent manner along with the decrease of the localization of Bub1 and Aurora B.

### Mis12-Bub1 WT and Mis12-SET promote Aurora B activation

**Figure 3:**
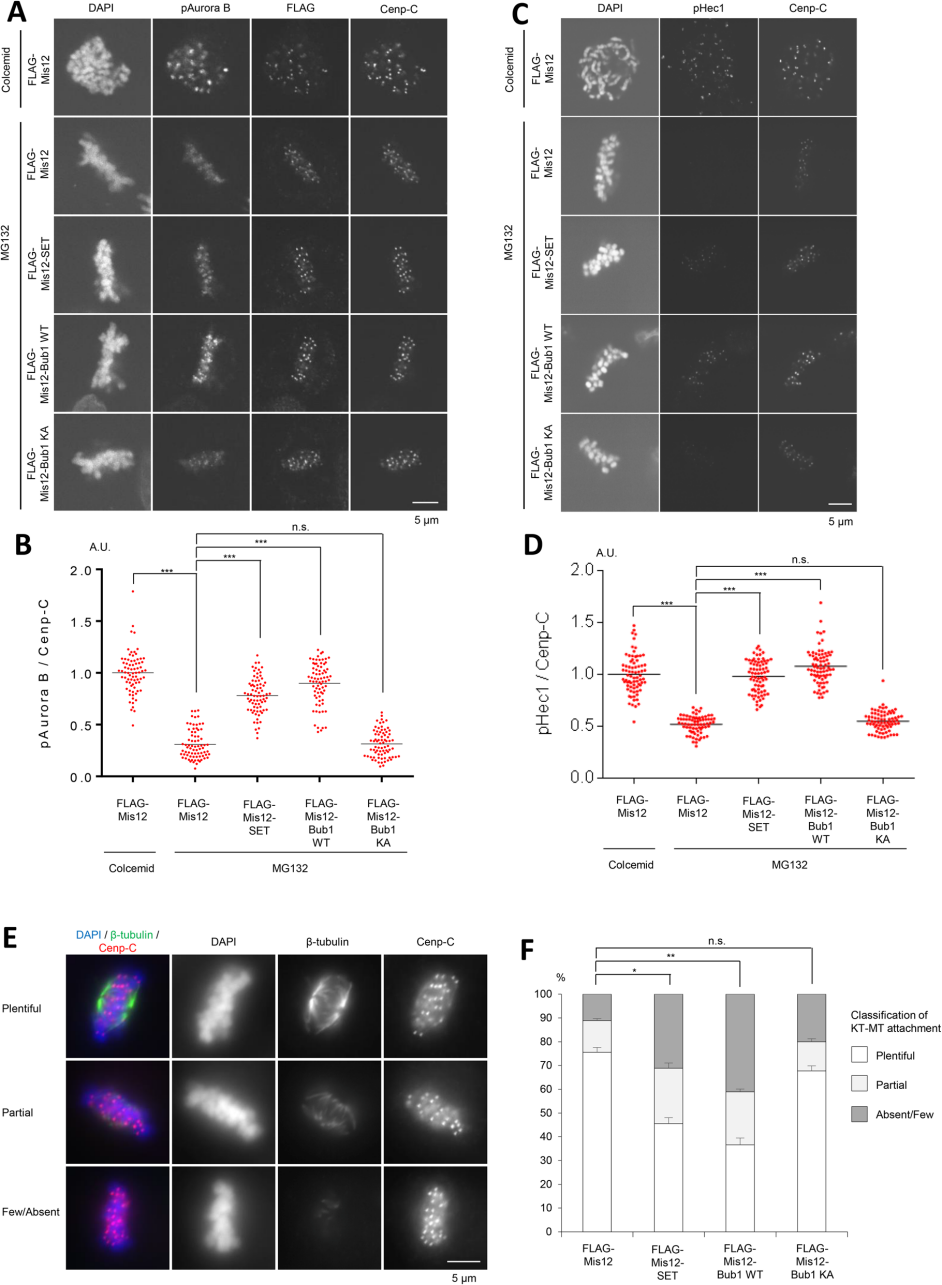

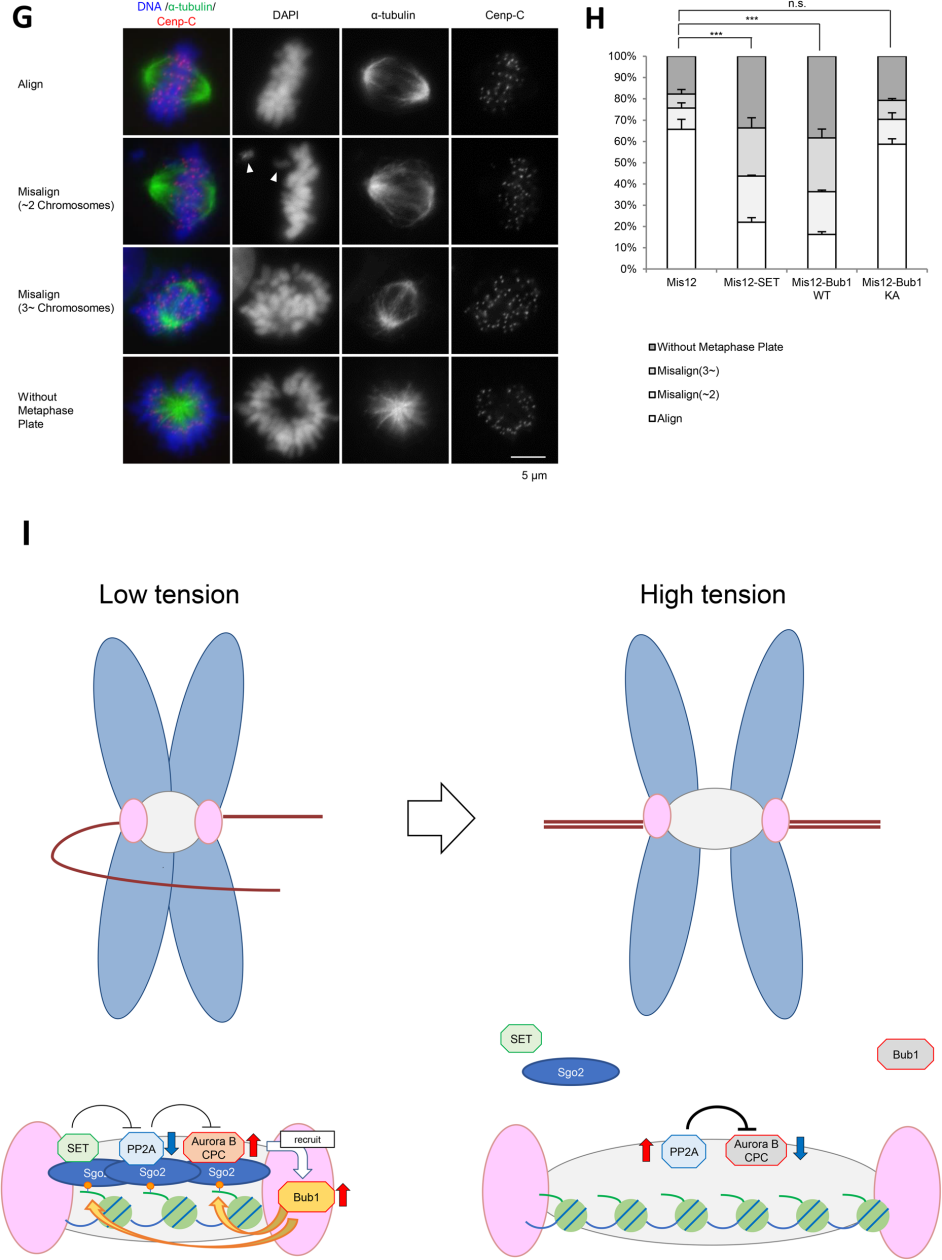
Expression of Mis12-SET bolsters Aurora B kinase at the inner centromeres, leading to defect in chromosome alignment. (**A**, **B**) Expression of Mis12-SET at metaphase activates Aurora B kinase at centromeres. (**A**) HeLa cells expressing 3FLAG-HA-Mis12, 3FLAG-HA-Mis12-SET, 3FLAG-HA-Mis12-Bub1 WT or 3FLAG-HA-Mis12 KA were arrested at prometaphase by colcemid treatment or at metaphase by MG132 treatment, and fixed and stained with anti-FLAG, pAurora B and Cenp-C antibodies. Scale bar, 5 μm. (**B**) Relative intensities (pAurora B/Cenp-C) of (**A**) are shown. Bars represent means. Dot plot (N = 75 cells, 3 independent experiment, 5 cells per experiment, 5 kinetochore pairs per cell, Mann–Whitney U-test, ***p < 0.001, n.s. not significant). (**C**, **D**) Expression of Mis12-SET at metaphase increases pHec1 signals at kinetochores. (**C**) HeLa cells prepared as for (**A**) were fixed and stained with anti-FLAG, pHec1 and Cenp-C antibodies. Scale bar, 5 μm. (**D**) Relative intensities (pHec1/Cenp-C) of (**C**) are shown. Bars represent means. Dot plot (N = 75 cells, 3 independent experiment, 5 cells per experiment, 5 kinetochore pairs per cell, Mann–Whitney U-test, ***p < 0.001, n.s. not significant). (**E**, **F**) HeLa cells expressing 3FLAG-HA-Mis12, 3FLAG-HA-Mis12-SET, 3FLAG-HA-Mis12-Bub1 WT or 3FLAG-HA-Mis12 KA were released from RO3306, and treated with MG132 for 90 min. Cells were then incubated in cold DMEM for 20 min, fixed and stained with anti-β-tubulin and Cenp-C antibodies. Scale bar, 5 μm. (**F**) Graph shows the frequency of metaphase cells with plentiful, partial or absent/few KT-MT attachments in (**E**). Bars represent SD (N = 3 independent experiments, 100 mitotic cells per experiment, Student’s t-tests were performed on the category “Plentiful”, *p < 0.05, **p < 0.01, n.s. not significant). (**G**, **H**) Expression of Mis12-SET at metaphase induces chromosome mis-alignment. (**G**) HeLa cells expressing 3FLAG-HA-Mis12, 3FLAG-HA-Mis12-SET, 3FLAG-HA-Mis12-Bub1 WT or 3FLAG-HA-Mis12 KA were released from RO3306, which arrests cells at G2/M transition, treated with MG132 for 60 min, and fixed and stained with anti-α-tubulin and Cenp-C antibodies. The white arrows indicate mis-aligned chromosomes. Scale bar, 5 μm. (**H**) Graph shows the frequency of mitotic cells with aligned chromosomes, 1 or 2 mis-aligned chromosomes, 3 or more mis-aligned chromosomes, or without the metaphase plate of (**G**). Bars represent SD (N = 3 independent experiments, 100 mitotic cells per experiment, Student’s t-tests were performed on the category “Align”, ***p < 0.001, n.s. not significant). (**I**) The model described in this article. Aurora B, Bub1 and SET form a kinetochore distance-dependent feedback loop for accurate chromosome alignment and proper chromosome separation. In prometaphase, Aurora B is enriched at the centromeres and activated through SET-mediated PP2A inhibition to correct improper KT-MT attachment. In contrast, during metaphase, the centromeric localization of SET decreases in a kinetochore distance-dependent manner, leading to PP2A activation and Aurora B inactivation for the stabilization of bi-oriented attachments.

Previous reports have shown that Bub1-phosphorylated HH2A T120 in centromeric nucleosomes generates a platform for the Aurora B adaptor protein, Sgo, and that Aurora B and Bub1 influence the localization of one another^17,19–21,30^. The foregoing results and several reports suggest the existence of a distant-dependent feedback loop between Aurora B and Bub1. Indeed, the expression of Mis12-Bub1 WT, but not KA, increased the autophosphorylation level of Aurora B at Thr232 (pAurora B) (Figs. 3A,B, S4A,B), and the phosphorylation levels of Hec1 (pHec1) (Fig. 3C,D). These results indicate that Bub1 enriched at the kinetochore remotely promotes the accumulation of Aurora B in a kinetochore distance-dependent manner.

In a previous report, it was suggested that a timely decrease of SET at the centromere in metaphase promotes proper chromosomal alignment by activating PP2A and inhibiting Aurora B kinase activity^40^. To evaluate the significance of the decrease of SET at the centromere in metaphase, kinetochore-tethered Mis12-SET fusion protein were expressed in metaphase-arrested cells in which most of the endogenous SET protein dissociates from centromeres. Like Mis12-Bub1 WT, Mis12-SET also increased Aurora B kinase activity (Fig. 3A–D). To investigate whether the increase in Aurora B kinase activity is induced by the activation of PP2A, we next planned to express a Mis12-SET mutant that cannot bind to PP2A as a negative control. Even though it has been reported that the N-terminus of SET with a V92A mutation loses its PP2Ac binding ability^48^, the binding assay showed the SET 1–119 aa V92A mutant still bound to PP2A (Fig. S4C). For this reason, instead, metaphase cells expressing Mis12-SET were treated with FTY720, a chemical inhibitor of the interaction between SET and PP2A^49^. FTY720 treatment reduced the phosphorylation on Hec1 by Aurora B kinase, which was once induced by the expression of Mis12-SET (Fig. S4D,E), suggesting that Mis12-SET maintains Aurora B kinase activity by inhibiting PP2A phosphatase activity. In addition, like Mis12-Bub1 WT, Mis12-SET expression induced both the destabilization of KT-MT attachment and the chromosomal mis-alignment in metaphase (Fig. 3E–H). These results strongly suggest our model in which SET maintains Aurora B kinase activity in prometaphase, and the decrease of centromeric localization of SET induces both PP2A activation and Aurora B kinase reduction for the formation of stable KT-MT attachment and chromosomal alignment in metaphase.

## Discussion

In prometaphase, Aurora B enriched at the inner centromeres is highly activated to destabilize any erroneously attached microtubules^2–4^, whereas the opposing phosphatase PP2A dephosphorylates the kinetochore substrates to stabilize KT-MT attachment. In metaphase, however, the geometry model proposes that bi-orientation pulls the kinetochores away from the Aurora B kinase zone, so that Aurora B cannot reach and phosphorylate the kinetochore^3,4,15^. Additionally, the amount of Aurora B and its kinase activity have been reported to decrease quickly between stretched and unstretched kinetochores^17,18^, suggesting that some unknown mechanism regulating Aurora B localization might be involved in tension sensing. In contrast, PP2A-B56 is distributed from the centromeres to the kinetochores, even when the tension becomes high in metaphase, leading to the establishment of chromosome bi-orientation^5,16^. However, it was not well understood how Aurora B kinase and PP2A activities are regulated from prometaphase to metaphase.

In a previous report, we showed that SET localizes at the centromeres on Sgo2, and up-regulates Aurora B kinase via PP2A inhibition^40^. Furthermore, centromeric localizations of SET and Sgo2 decrease in a sister kinetochore distance-dependent manner, indicating that the decrease might promote PP2A activation and Aurora B inactivation to stabilize proper KT-MT attachment in metaphase.

Our results here show that kinetochore-associated Bub1 and inner centromere Aurora B remotely enhance the localization of one another in a kinetochore distance-dependent manner (Figs. 1E–N, 3A,B, S1A–D). In addition, the localization of both SET and Sgo2 localization at centromeres are greatly decreased in both Bub1-depleted and Aurora B-inhibited cells (Fig. 2F–H). These results suggest that SET and Sgo2 are involved in an Aurora B-Bub1 kinase feedback loop by maintaining Aurora B kinase activity. Indeed, the expression of kinetochore-tethered Mis12-Bub1 WT dramatically restored both SET and Sgo2 signals at centromeres in metaphase-arrested cells (Fig. 2I–N). Our analyses further show that the decrease in the centromeric localizations of SET and Sgo2 attenuates the positive feedback loop between Aurora B and Bub1 in a sister kinetochore distance-dependent manner. Indeed, the expression of Mis12-SET greatly restored Aurora B kinase activity, leading to chromosome mis-alignment (Fig. 3A–H). These results suggest that SET on Sgo2 also forms a positive feedback loop with Aurora B and Bub1 in a sister kinetochore distance-dependent manner. Finally, the restoration of Aurora B kinase activity by Mis12-SET is cancelled by treatment with FTY720, a SET inhibitor^49^, indicating that the decrease in the centromeric localization of SET in metaphase is required for dephosphorylation at the kinetochore and KT-MT attachment through PP2A activation (Fig. S4D,E). We thus propose that Aurora B, Bub1 and SET form a kinetochore distance-dependent positive feedback loop that activates Aurora B for the correction of improper KT-MT attachment, whereas the kinetochore distance-dependent decrease of centromeric SET ensures the timely formation of bi-orientation by inversely fine-tuning both the Aurora B kinase and PP2A phosphatase activities (Fig. 3I).

## Materials and methods

### Antibodies

The following antibodies were used for immunofluorescence and immunoblot: Anti-Mad2 (sc-65492, Santa Cruz Biotechnology, IF 1:75), Bub1 (K0168-3, MBL, IF 1:100, IB 1:1,000), CAP-H (sc-101013, Santa Cruz Biotechnologies, IB 1:1,000), Histone H2A phospho Thr 120^32^ (IF 1:50), FLAG (012-22384, Wako, IF 1:1,000 or F7425, Sigma, 1:500), SET/TAF1β^50^ (KM1720, IF 1:50, IB 1:2,000), hSgo2^51^ (IF 1:2,000 for Fig. 2A and 1:5,000 for Fig. 2F and L, IB 1:1,000), Aurora B (611,082, BD, IF 1:100, IB 1:1,000), pAurora A (T288)/B (T232) (2914S, Cell Signaling, IF 1:50, IB 1:2,000), Hec1 phospho Ser 55 (GTX70017, Gene Tex, IF 1:10,000), cyclin B1 (sc-245, Santa Cruz Biotechnology, IB 1:500), α-tubulin (T9026, Sigma, IF 1:5,000, IB 1:10,000), β-tubulin (T4026, Sigma, IF 1:1,000), GST (sc-459, Santa Cruz Biotechnology, IB 1:1,000) and Cenp-C (PD030, MBL, IF 1:1,000) antibodies. A For immunofluorescence, secondary antibodies (Invitrogen Alexa Fluor) were used at 1:1,000. For immunoblotting, secondary antibodies were used at 1:2,000.

### Plasmid construction

Mis12 and Bub1 were amplified by PCR from cDNA of HeLa cells, and SETβ was amplified by PCR from pCAGGS/HA-SETβ^40^. The PCR products were cloned into the Xho1 site of pInducer20 by InFusion. For Bub1 K821A mutation, inverse PCR was performed with the following primers; sense: TTTTAGCGGTCCAAAAGCCTGCCAACCCCT, antisense: TTGGACCGCTAAAACAAATTTCTGTTTATT.

### Cell culture and treatment

293 T cell line were obtained from the American Type Culture Collection (ATCC). The HeLa-1 Chromosomal instability (CIN^-^) cell line was selected as a clone in which chromosome segregation errors are less frequent (provided by M. Ohsugi^52^). Cells were cultured in Dulbecco's Modified Eagle’s Medium (DMEM, Gibco) with 10% FBS, and penicillin–streptomycin (1:100, Wako) at 37 °C in the presence of 5% CO_2_. For cell synchronization, nocodazole (Calbiochem), colcemid (gibco), RO3306 (Sigma) and MG132 were used at 100 ng/ml, 100 ng/ml, 6 µM and 10 µM, respectively. For Aurora B inhibition, ZM447439 (AstraZeneca) or AZD1152 (Sellechem) was used at 10 µM, respectively. The cells were selected with 1 ~ 10 ng/ml puromycin. Doxycycline (Sigma) was used at 2 μg/mL for induction of protein expression from pInducer.

### siRNA transfection

Synthetic siRNA duplexes of CAP-H, Bub1 or Luciferase (control) were transfected into HeLa cells with the transfection reagent Lipofectamine RNAi max (Invitrogen). siRNA sequences are as follows:

- siCAP-H sense: cauuacuccaccuguaucaTT,
- siCAP-H antisense: tgatacaggtggagtaatgTT^42^,
- siBub1 #1 sense: cccauuugccagcucaagcTT,
- siBub1 #1 antisense: gcuugagcuggcaaaugggTT,
- siBub1 #2 sense: ccagugaguuccuauccaaTT,
- siBub1 #2 antisense: uuggauaggaacucacuggTT^30^,
- siLuciferase sense: cguacgcggaauacuucgaTT,
- siLuciferase antisense: ucgaaguauuccgcguacgTT.

For depletion of CAP-H, 160 nM siCAP-H was transfected. For depletion of Bub1, 40 nM siBub1 #1 and 40 nM siBub1 #2 were transfected twice.

### Generation of lentivirus

For generation of lentivirus, pInducer/3FLAG-HA-Mis12, pInducer/3FLAG-HA-Mis12-Bub1 WT, pInducer/3FLAG-HA-Mis12-Bub1 K821A or pInducer/3FLAG-HA-Mis12-SETβ were co-transfected with pMD2G (addgene) and psPAX2 (addgene) to 293 T cell with polyethelenimin, and cultured for at least 2 days. Lentiviruses were collected and used to infect HeLa cells.

### Immunofluorescence and microscopy

For immunofluorescence microscopy of Mad2, Bub1, SETβ, Sgo2 or pAurora B, cells cultured on coverslips with Cell-Tak (CORNING) were treated with 0.2% TritonX-100 in PBS for 2 min before fixation with 1% PFA-0.2% TritonX-100 in PBS for 10 min. For analysis of phosphorylation on Hec1 S55, cells cultured on coverslips were incubated in × 4 PBS at 37 °C for 20 min before fixation with 1% PFA-0.2% TritonX-100 in PBS for 10 min. For analysis of α-tubulin, cells cultured on coverslips were fixed with 1% PFA-0.2% TritonX-100 in PBS for 10 min. After the fixation, cells were treated with 0.2% TritonX-100 in PBS for 10 min, blocked with 3% BSA in PBS for 15 min, incubated with primary antibodies diluted in 3% BSA in PBS overnight at 4 °C, and then incubated with secondary antibodies at r.t. for 1 h. Finally, DNA was stained with 3 uM DAPI (Sigma). Analysis of immunofluorescence was performed on a fluorescence microscope ECLIPSE TE2000-E (NIKON) or FV-1000 (Olympus) with a × 100 objective.

### Measurement of distances between Cenp-C signals

To measure the distances between Cenp-C signals, 2D images obtained on a confocal microscope FV-1000 (Olympus) were used without stacking. First, the 2D images were opened with ImageJ software (ImageJ bundled with 64-bit Java 1.8.0_112; https://imagej.nih.gov/ij/download.html), and four clearly paired Cenp-C signals were chosen and circled in each cell. Then, the “Center of the Mass”, the coordinate (x, y), of each Cenp-C signal was measured by the following plugin: “Analysis > Measure > Center of the Mass”. Finally, the distance (d) between paired Cenp-C signals (x1, y1) and (x2, y2) was calculated by the following formula utilizing the “Pythagorean theorem”, using Microsoft Excel (Microsoft Office Home and Business 2019; https://www.microsoft.com/ja-jp/microsoft-365/microsoft-office?rtc=1):

$$ d = \left\{ {(x1 - x2)^{2} + (y1 - y2)^{2} } \right\}^{0.5} . $$

### Quantification of signals at centromeres and kinetochores

Signals at the kinetochores were quantified using ImageJ software as follows. First, the gray scale 2D images of each color were opened with ImageJ software, and clearly paired Cenp-C signals were chosen in each cell. Next, the kinetochores or centromeres were circled, and then the “Mean gray value” of every circle was measured by the following plugin: “Analysis > Measure > Mean gray value”. Finally, every Mean gray value was divided by the value of the corresponding Cenp-C.

### Combining distances between Cenp-C signals, and quantification of signals at centromeres and kinetochores

Quantification of signals at centromeres and kinetochores was performed as in the section “Quantification of signals at centromeres and kinetochores”, and the distances between corresponding Cenp-C signals were measured as in the section “Measurement of distances between Cenp-C signals”. The quantified amounts were plotted against the measured distances on the scatter plot of Microsoft Excel.

### Trypan blue assay

PBS with 0.4% Trypan blue and DMEM with cells were combined at a ratio 1:1, and then the ratios of stained cells were calculated.

### Purifications of GST-tagged proteins

BL21 cells transformed with pGEX/4T-2, pGEX/SET 1–119 aa, or pGEX/SET 1–119 aa V92A were cultured, and the expressions were induced by IPTG treatment. Next, BL21 cells were collected and suspended in extraction buffer (20 mM Tris–HCl at pH 7.5, 150 mM NaCl, 1% TritonX-100, protein inhibitor cocktail (Nacalai Tesque). Then, Glutathione Sepharose 4B (Sigma) was added and rotated for 1 h, and the beads were washed with extraction buffer. Finally, GST-tagged SET mutants were eluted using elution buffer [100 mM Tris–HCl at pH 8.0, 150 mM NaCl, 20 mM glutathione (reduced form)].

### Pull-down assay

293 T cells transfected with pCMV/FLAG-PP2Ac were collected and suspended in extraction buffer [20 mM Tris–HCl at pH 7.5, 150 mM NaCl, 1% TritonX-100, protein inhibitor cocktail, PhosStop (Sigma)]. Next, the lysates were added to Glutathione Sepharose 4B with GST-tagged proteins, and rotated for 2 h. Then, the beads were washed with extraction buffer, and proteins were detected by western blot.

### Statistical analysis

Statistical significance was analyzed by the Mann–Whitney *U*-test or Student’s t-test using Microsoft Excel and GraphPad Prism version 6.03 (GraphPad Software; https://www.mdf-soft.com/). Additionally, ANOVA analysis followed by Tukey’s multiple comparison tests were also performed using GraphPad Prism version 6.03 and Microsoft Excel, respectively. The results of ANOVA analysis and Tukey’s multiple comparison tests were consistent with those of the Mann–Whitney *U*-tests and Student’s t-test.

## Supplementary information

Supplementary Legend.

Supplementary Figures.
