# Supplementary material for: SET/TAF1 forms a distance-dependent feedback loop with Aurora B and Bub1 as a tension sensor at centromeres
Source: Sci Rep. 2020 Sep 24;10:15653. doi: 10.1038/s41598-020-71955-2 (PMC7518443; doi:10.1038/s41598-020-71955-2)
Supplement: Supplementary file 1 — Supplementary Legend. [file 41598_2020_71955_MOESM1_ESM.docx]

**SET/TAF1 forms a distance-dependent feedback loop with Aurora B and Bub1 as a tension sensor at centromeres**

Yuichiro Asai^1^, Rieko Matsumura^1^, Yurina Hasumi^1^, Hiroaki Susumu^2^, Kyosuke Nagata^3^, Yoshinori Watanabe^4^, and Yasuhiko Terada^1,*^

^1^Department of Chemistry and Biochemistry, School of Advanced Science and Engineering, Waseda University, 3-4-1 Ohkubo, Shinjuku-ku Tokyo 169-8555, Japan

^2^Graduate Program in Biophysics and Biochemistry, Graduate School of Science, The University of Tokyo, Yayoi, Tokyo 113-0032, Japan

^3^Department of Infection Biology, Faculty of Medicine, University of Tsukuba, 1-1-1 Tennodai, Tsukuba 305-8575, Japan.

^4^Genome Damage and Stability Centre, School of Life Sciences, University of Sussex, Falmer, Sussex, BN1 9RQ, UK.

^*^Correspondence should be addressed to Y.T. (e-mail: [yterada@waseda.jp](mailto:yterada@waseda.jp))

Telephone: +81-3-5286-3307 FAX: +81-3-5286-3307

Running Title: A distance-dependent feedback loop as a tension sensor at the centromere

**Figure legends for Supplemental figures**

**Figure S1**. **Aurora B kinase activity is required for Bub1 localization and its kinase activity.**

(**A**) HeLa cells arrested in prometaphase by nocodazole treatment were treated with Bub1 or control RNAi plus ZM447439 (Aurora kinases inhibitor), AZD1152 (Aurora B kinase inhibitor) or DMSO (control), collected by mitotic shake off, and then analyzed by immunoblot with the indicated antibodies.

(**B-D**) HeLa cells arrested in prometaphase by colcemid treatment were treated as in (**A**), and stained with anti-Bub1, HH2A pT120 and Cenp-C antibodies. Scale bar, 5 μm. Bars represent means. Dot plot (N = 25 kinetochore pairs from 5 cells, 5 kinetochore pairs per cell, Mann-Whitney U-test, ***p<0.001).

**Figure S2. Kinetochore distances were artificially changed by CAPH RNAi and taxol treatment.**

(**A**) HeLa cells treated with the RNAi against CAPH (in the range of 40 to 320 nM) or Luciferase (control) were analyzed by immunoblot with anti-CAPH or anti-α-tubulin antibodies. The CAPH membrane was cropped to exclude the nonspecific bands. (**B**) Cells treated as in (**A**) were collected and stained with trypan blue. Relative rates of trypan blue positive cells are shown. In all experiments, cells were transfected with 160 nM RNAi against CAPH due to its low toxicity.

(**C and D**) For Fig. 1 and Fig. 2A-E, kinetochore distances of HeLa cells were altered as shown.

**Figure S3**. **Expression of Mis12-Bub1 at metaphase restores HH2A pT120 signals at kinetochores.**

(**A**) HeLa cells expressing 3FLAG-HA-Mis12, 3FLAG-HA-Mis12-Bub1 WT or 3FLAG-HA-Mis12 KA (kinase dead mutant) were arrested at prometaphase by colcemid treatment or at metaphase by MG132 treatment, and fixed and stained with anti-FLAG, HH2A pT120 and Cenp-C antibodies. Scale bar, 5 μm. (**B and D**) Relative intensities (FLAG / Cenp-C or HH2A pT120 / Cenp-C) of (**A)** are shown. Bars represent means. Dot plot (N = 75 kinetochore pairs, 3 independent experiment, 5 cells per experiment, 5 kinetochore pairs per cell, Mann-Whitney U-test, ***p<0.001, n.s. not significant). (**C**) Distances between paired kinetochores (kinetochore-distances) of (**A**) are shown. Dot plot (N = 75 kinetochore pairs, 3 independent experiment, 5 cells per experiment, 5 kinetochore pairs per cell, Mann-Whitney U-test, ***p<0.001, n.s. not significant).

**Figure S4. Ectopic kinetochore localization of SET activates Aurora B by PP2A inhibition at metaphase.**

(**A and B**) Relative intensities (FLAG / Cenp-C) or kinetochore-distances of **Fig. 3A** are shown. Dot plots (N = 75 cells, 3 independent experiment, 5 kinetochore pairs per cell, 5 cells per experiment, Mann-Whitney U-test, ***p<0.001, n.s. not significant).

(**C**) 293T cells expressing FLAG-PP2Ac were collected and suspended in extraction buffer. The lysates were added to Glutathione Sepharose 4B with GST, GST-SETβ 1−119 or GST-SETβ 1−119 V92A, and rotated for 2 hr. Then, the beads were washed with extraction buffer, and proteins were detected by western blot.

(**D and E**) HeLa cells expressing 3FLAG-HA-Mis12 or 3FLAG-HA-Mis12-SET were arrested at prometaphase by colcemid treatment or at metaphase by MG132 treatment, treated with FTY720 (SET inhibitor that prevents the formation of SET/PP2Ac complex) ^32^ or pFTY720 (inactive form of FTY720) ^32^, and fixed and stained with anti-FLAG, pHec1 and Cenp-C antibodies. Relative intensities (FLAG / Cenp-C or pHec1 / Cenp-C) are shown. Bars represent means. Dot plot (N = 75 cells, 3 independent experiment, 5 cells per experiment, 5 kinetochore pairs per cell, Mann-Whitney U-test, ***p<0.001, n.s. not significant).

**Figure S5. Raw data.**

The raw data of Fig. S1A, Fig. S2A and Fig. S4C are shown.
