# Supplementary material for: SET/TAF1 forms a distance-dependent feedback loop with Aurora B and Bub1 as a tension sensor at centromeres
Source: Sci Rep. 2020 Sep 24;10:15653. doi: 10.1038/s41598-020-71955-2 (PMC7518443; doi:10.1038/s41598-020-71955-2)

Figure S1

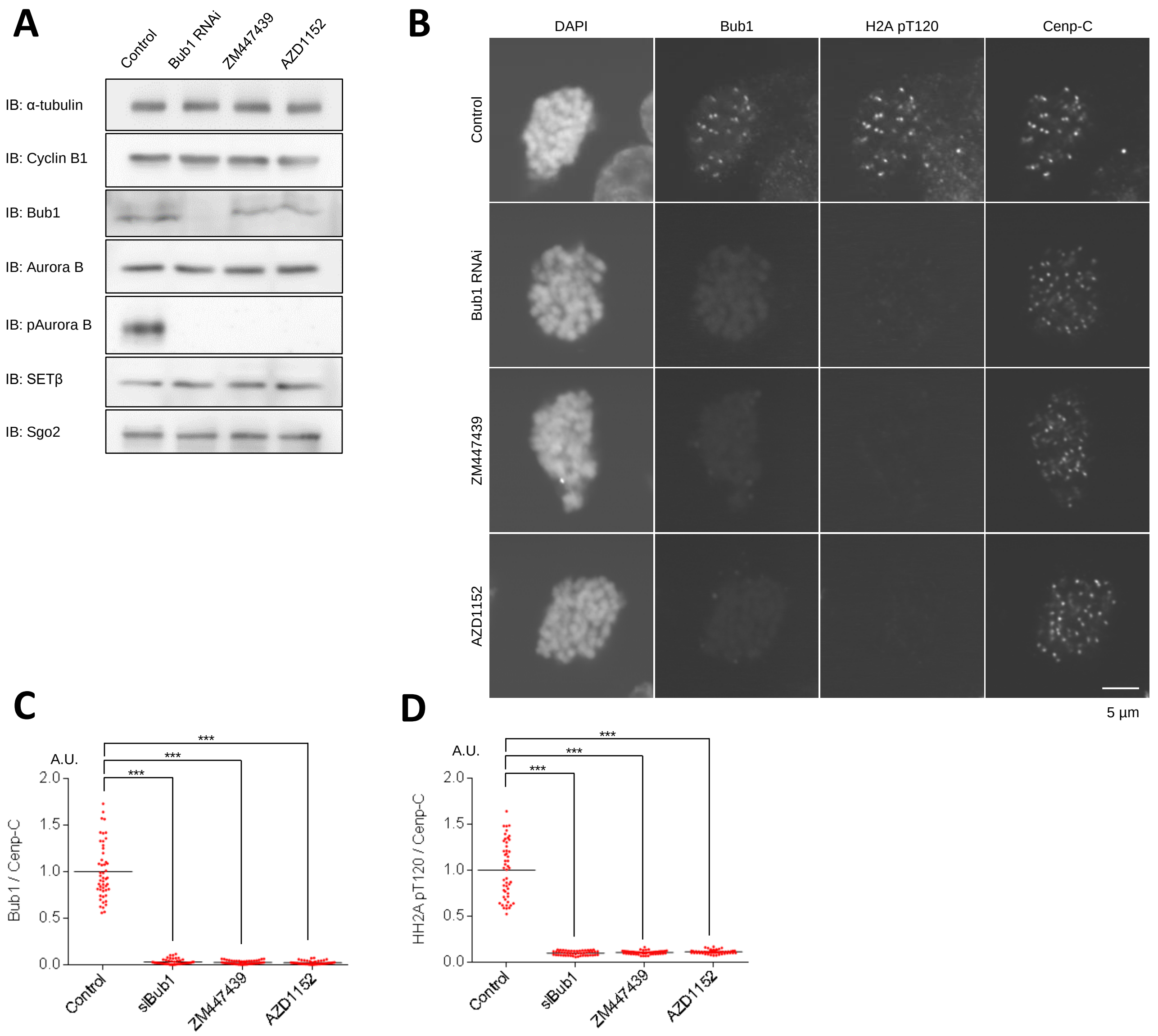
A
Control
Bub1 RNAi
ZM447439
AZD1152
IB: α-tubulin
IB: Cyclin B1
IB: Bub1
IB: Aurora B
IB: pAurora B
IB: SETβ
IB: Sgo2
B
DAPI
Bub1
H2A pT120
Cenp-C
Control
Bub1 RNAi
ZM447439
AZD1152
5 µm
C
A.U.
Bub1 / Cenp-C
***
2.0
1.5
1.0
0.5
0.0
Control
siBub1
ZM447439
AZD1152
D
A.U.
HH2A pT120 / Cenp-C
***
2.0
1.5
1.0
0.5
0.0
Control
siBub1
ZM447439
AZD1152

Figure S2

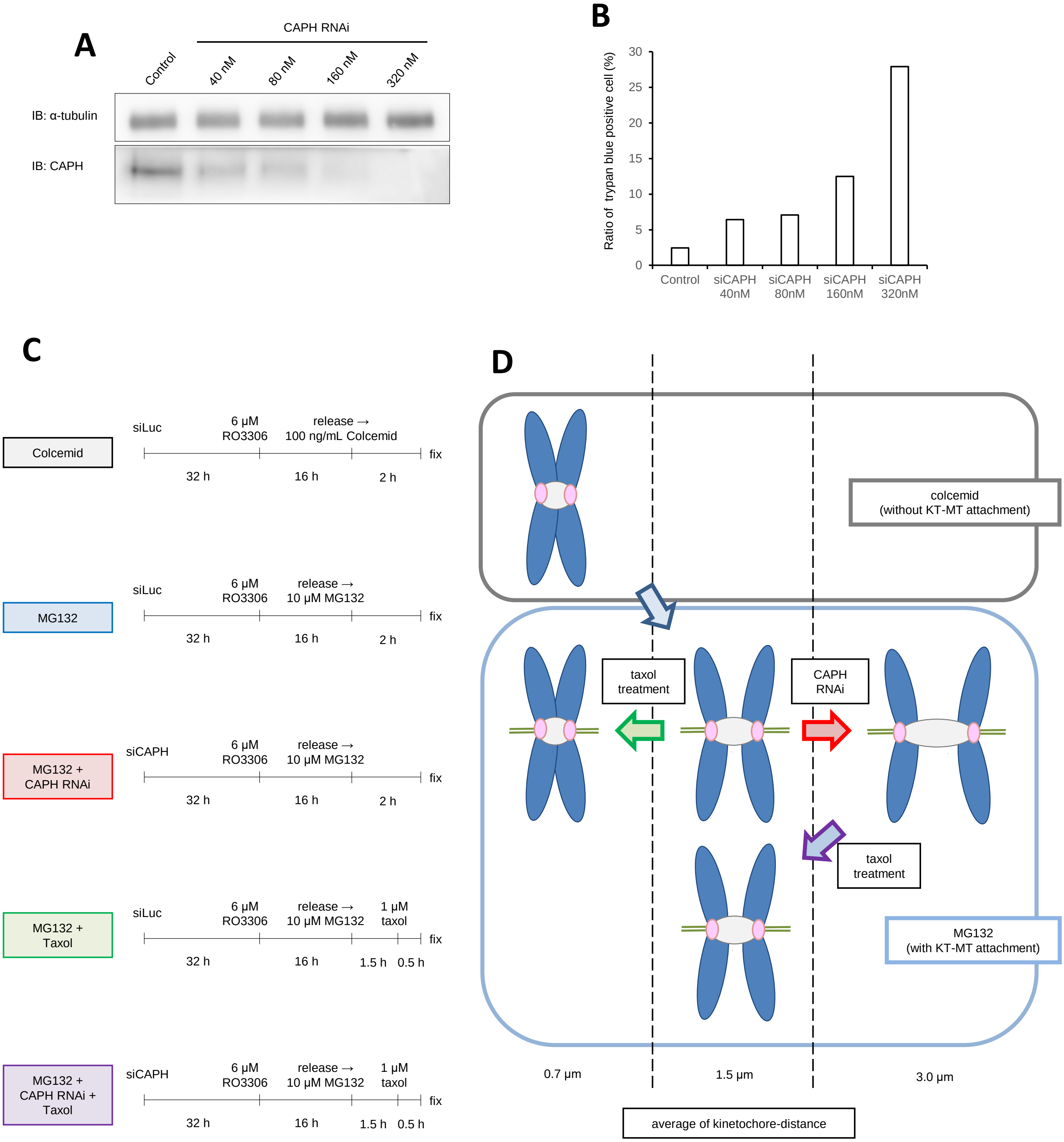

A
CAPH RNAi
Control
40 nM
80 nM
160 nM
320 nM
IB: α-tubulin
IB: CAPH
B
Ratio of trypan blue positive cell (%)
0
5
10
15
20
25
30
Control
siCAPH 40nM
siCAPH 80nM
siCAPH 160nM
siCAPH 320nM
C
Colcemid
siLuc
6 μM RO3306
release → 100 ng/mL Colcemid
fix
32 h
16 h
2 h
MG132
siLuc
6 μM RO3306
release → 10 μM MG132
fix
32 h
16 h
2 h
MG132 + CAPH RNAi
siCAPH
6 μM RO3306
release → 10 μM MG132
fix
32 h
16 h
2 h
MG132 + Taxol
siLuc
6 μM RO3306
release → 10 μM MG132
1 μM taxol
fix
32 h
16 h
1.5 h
0.5 h
MG132 + CAPH RNAi + Taxol
siCAPH
6 μM RO3306
release → 10 μM MG132
1 μM taxol
fix
32 h
16 h
1.5 h
0.5 h
D
colcemid (without KT-MT attachment)
taxol treatment
CAPH RNAi
taxol treatment
MG132 (with KT-MT attachment)
0.7 μm
1.5 μm
3.0 μm
average of kinetochore-distance

Figure S3

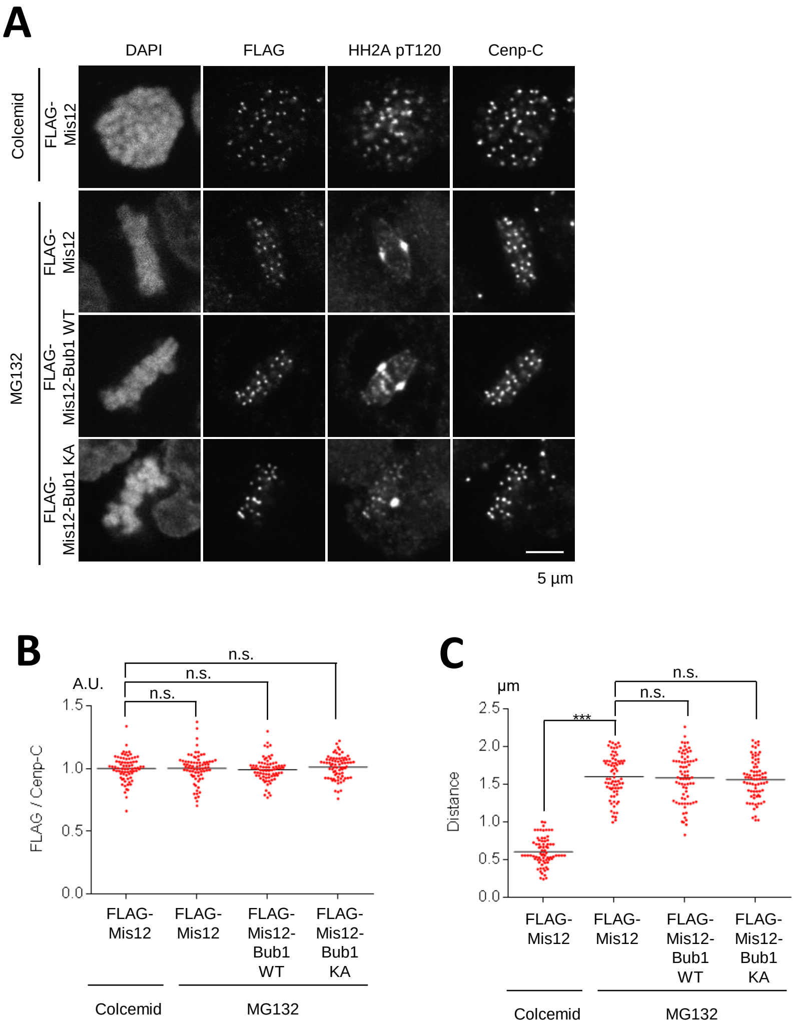
A
DAPI
FLAG
HH2A pT120
Cenp-C
Colcemid
FLAG-Mis12
MG132
FLAG-Mis12
FLAG-Mis12-Bub1 WT
FLAG-Mis12-Bub1 KA
5 µm
B
A.U.
n.s.
n.s.
n.s.
FLAG / Cenp-C
1.5
1.0
0.5
0.0
FLAG-Mis12
FLAG-Mis12
FLAG-Mis12-Bub1 WT
FLAG-Mis12-Bub1 KA
Colcemid
MG132
C
µm
n.s.
n.s.
***
Distance
2.5
2.0
1.5
1.0
0.5
0.0
FLAG-Mis12
FLAG-Mis12
FLAG-Mis12-Bub1 WT
FLAG-Mis12-Bub1 KA
Colcemid
MG132
D
A.U.
n.s.
***
***
HH2A pT120 / Cenp-C
2.0
1.5
1.0
0.5
0.0
FLAG-Mis12
FLAG-Mis12
FLAG-Mis12-Bub1 WT
FLAG-Mis12-Bub1 KA
Colcemid
MG132

Figure S4

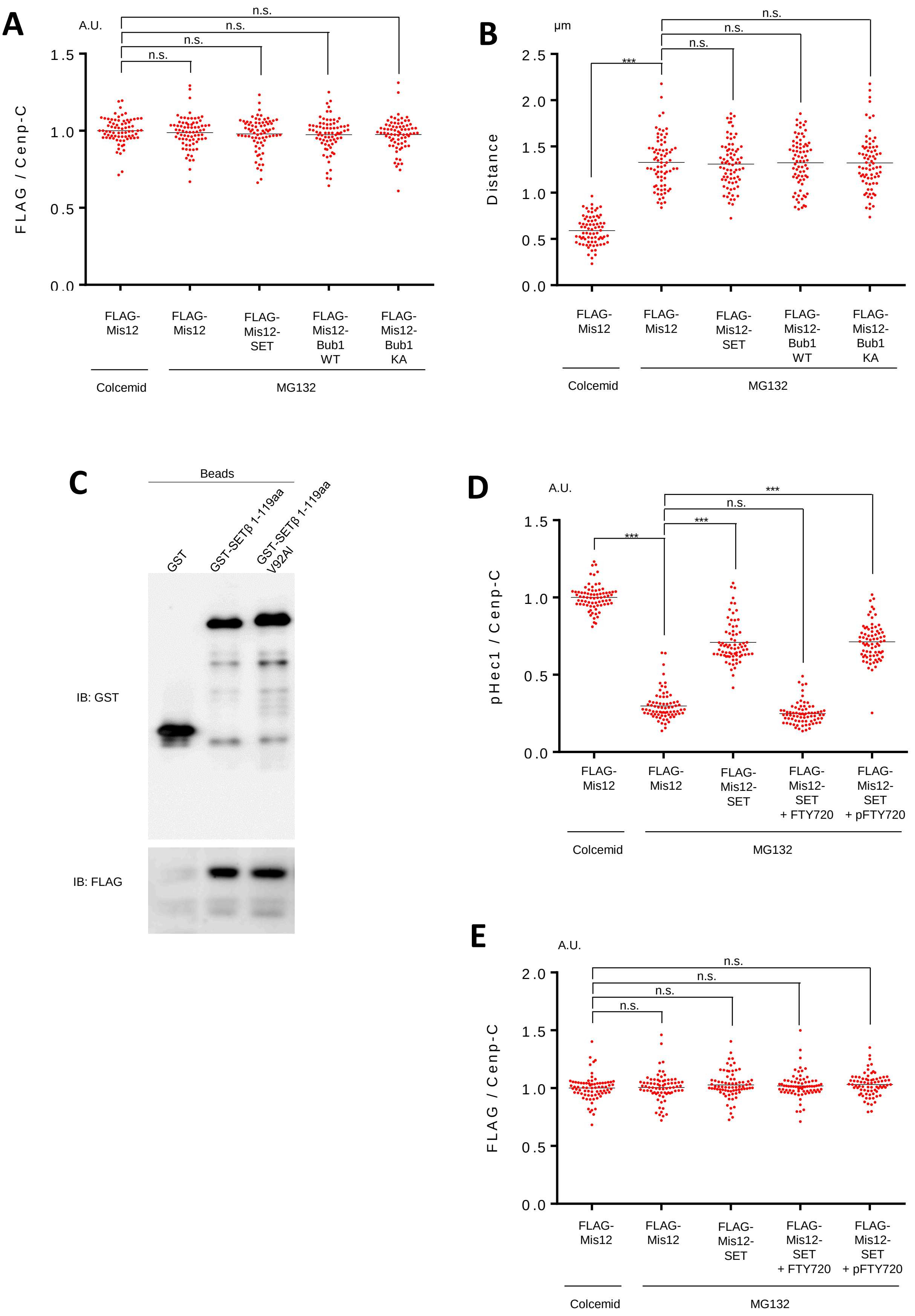

A
A.U.
FLAG / Cenp-C
n.s.
FLAG-Mis12
FLAG-Mis12-SET
FLAG-Mis12-Bub1 WT
FLAG-Mis12-Bub1 KA
Colcemid
MG132
B
μm
Distance
***
n.s.
C
Beads
GST
GST-SETβ 1-119aa
GST-SETβ 1-119aa V92AI
IB: GST
IB: FLAG
D
A.U.
pHec1 / Cenp-C
FLAG-Mis12-SET + FTY720
FLAG-Mis12-SET + pFTY720
E
A.U.
FLAG / Cenp-C

Figure S5

Raw data of Fig. S1A blots

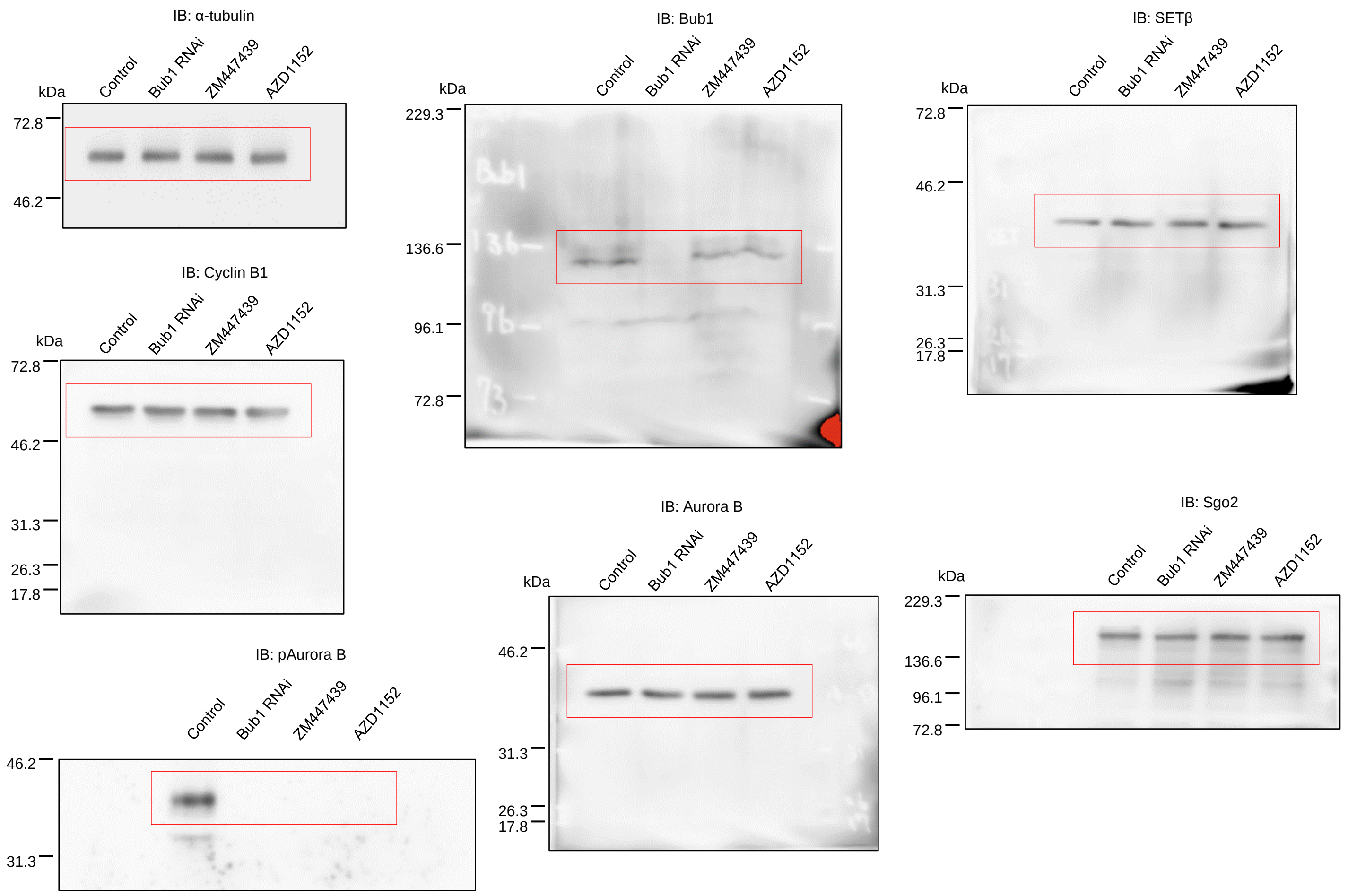

Raw data of Fig. S2A blots

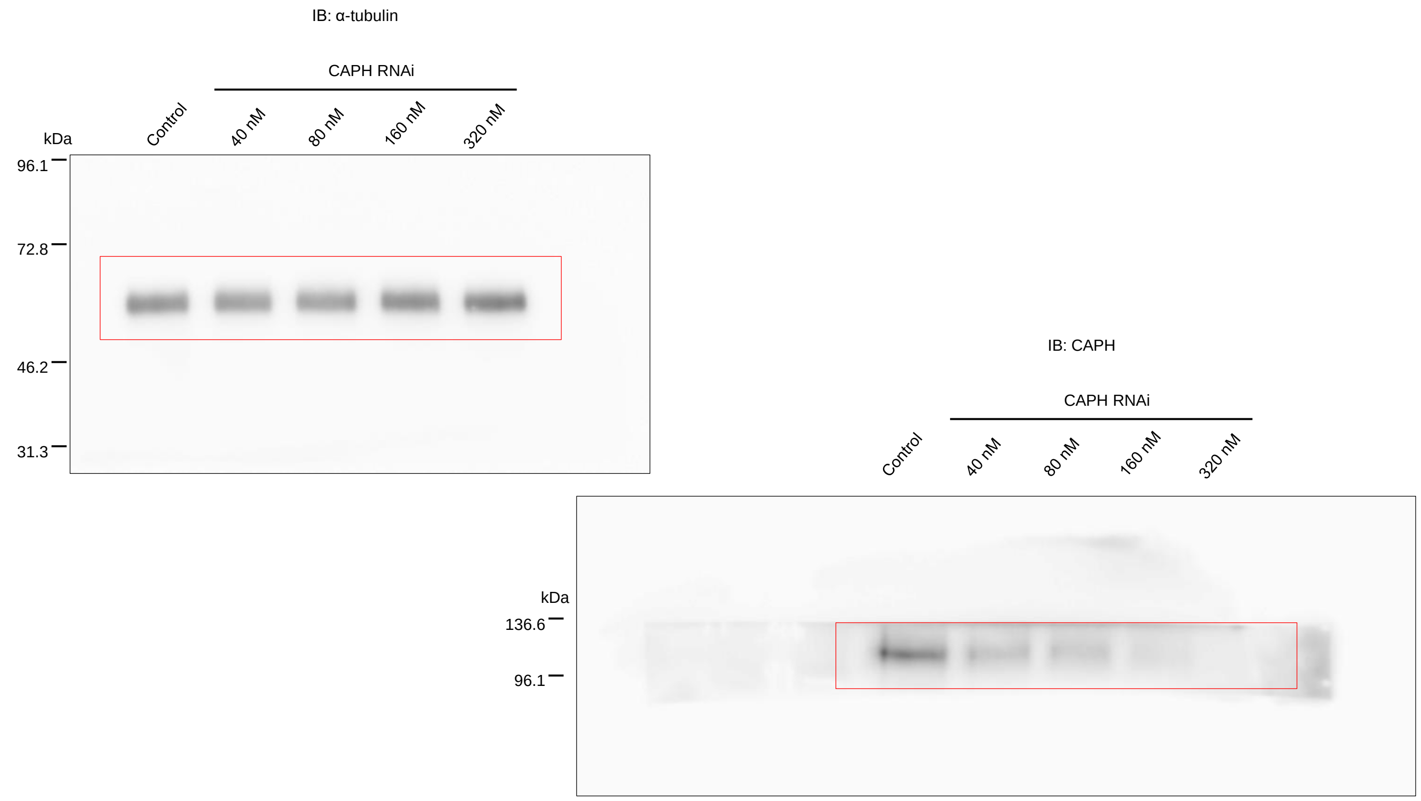

Raw data of Fig. S4C blots

IB: GST

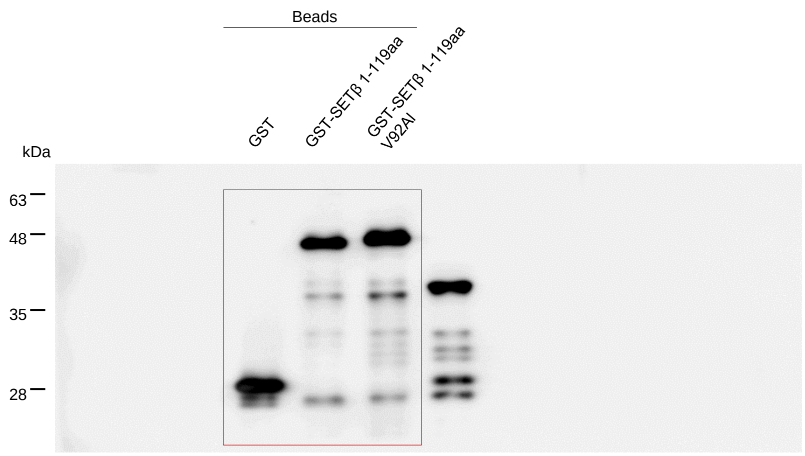

IB: FLAG

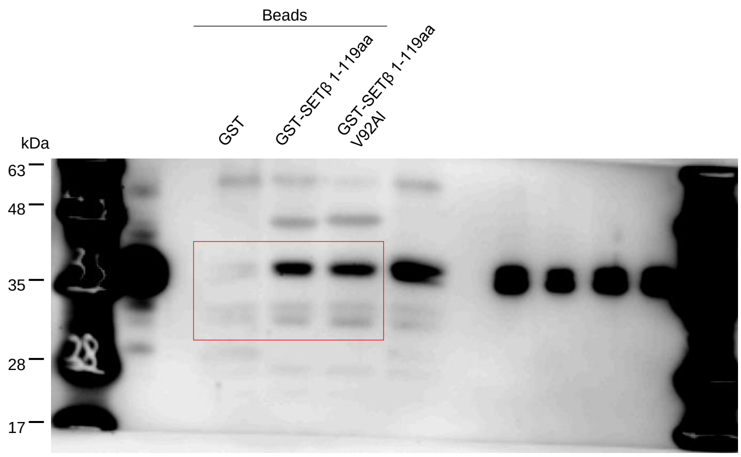

Supplement: Supplementary file 2 — Supplementary Figures. [file 41598_2020_71955_MOESM2_ESM.pdf]
